# Supplementary material for: Comparison of 2D and 3D oxygen-enhanced MRI of the placenta
Source: PLoS One. 2024 May 22;19(5):e0302623. doi: 10.1371/journal.pone.0302623 (PMC11111072; doi:10.1371/journal.pone.0302623)
Supplement: S4 File — Powerpoint file containing videos of ss-2D images and vol-3D videos of voxel-wise ΔR1 maps for all 12 pregnant subjects. (PPTX) [file pone.0302623.s004.pptx]

## Slide 1
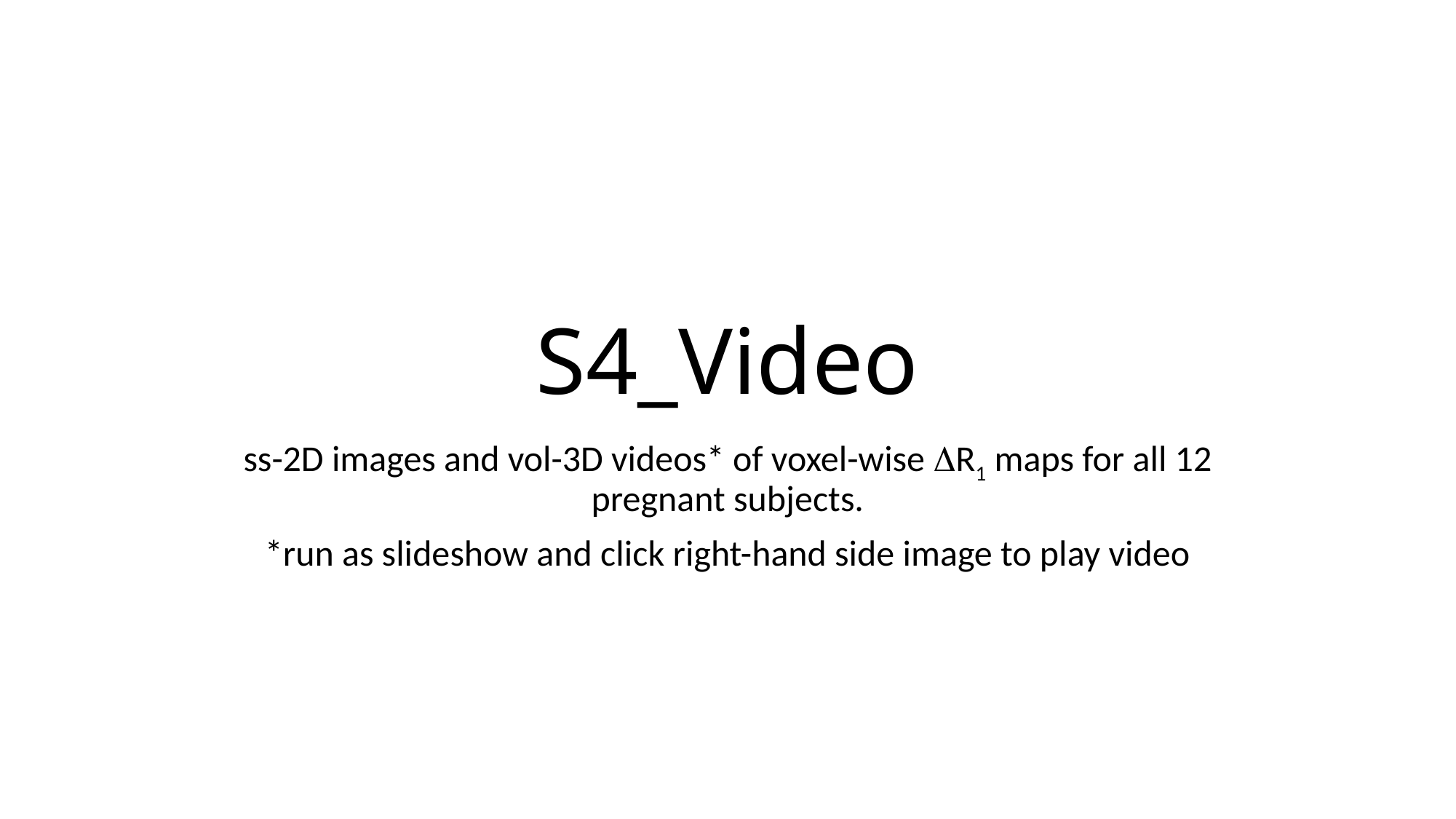

# S4_Video
ss-2D images and vol-3D videos* of voxel-wise DR1 maps for all 12 pregnant subjects.
*run as slideshow and click right-hand side image to play video

## Slide 2
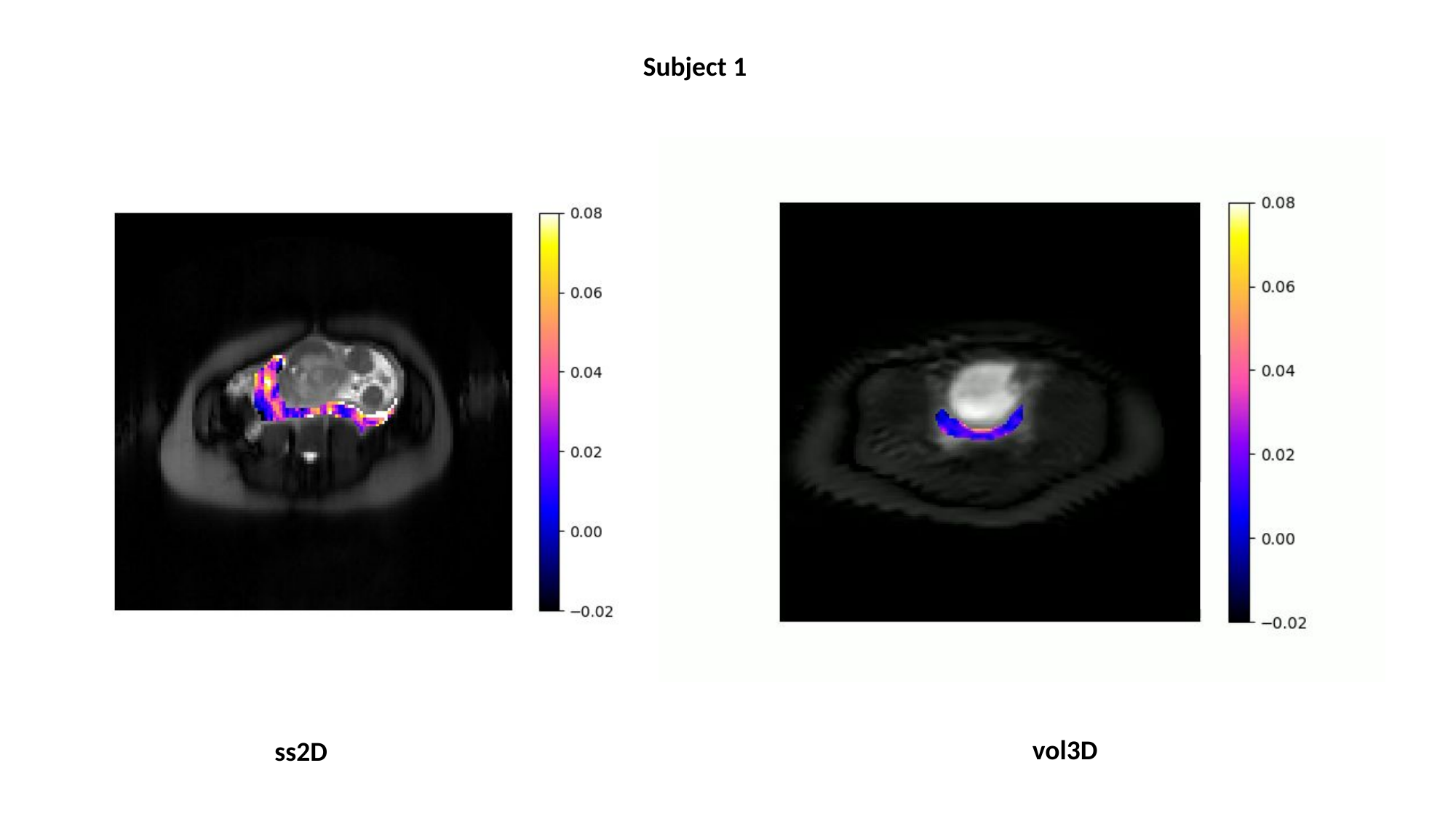

Subject 1
vol3D
ss2D

## Slide 3
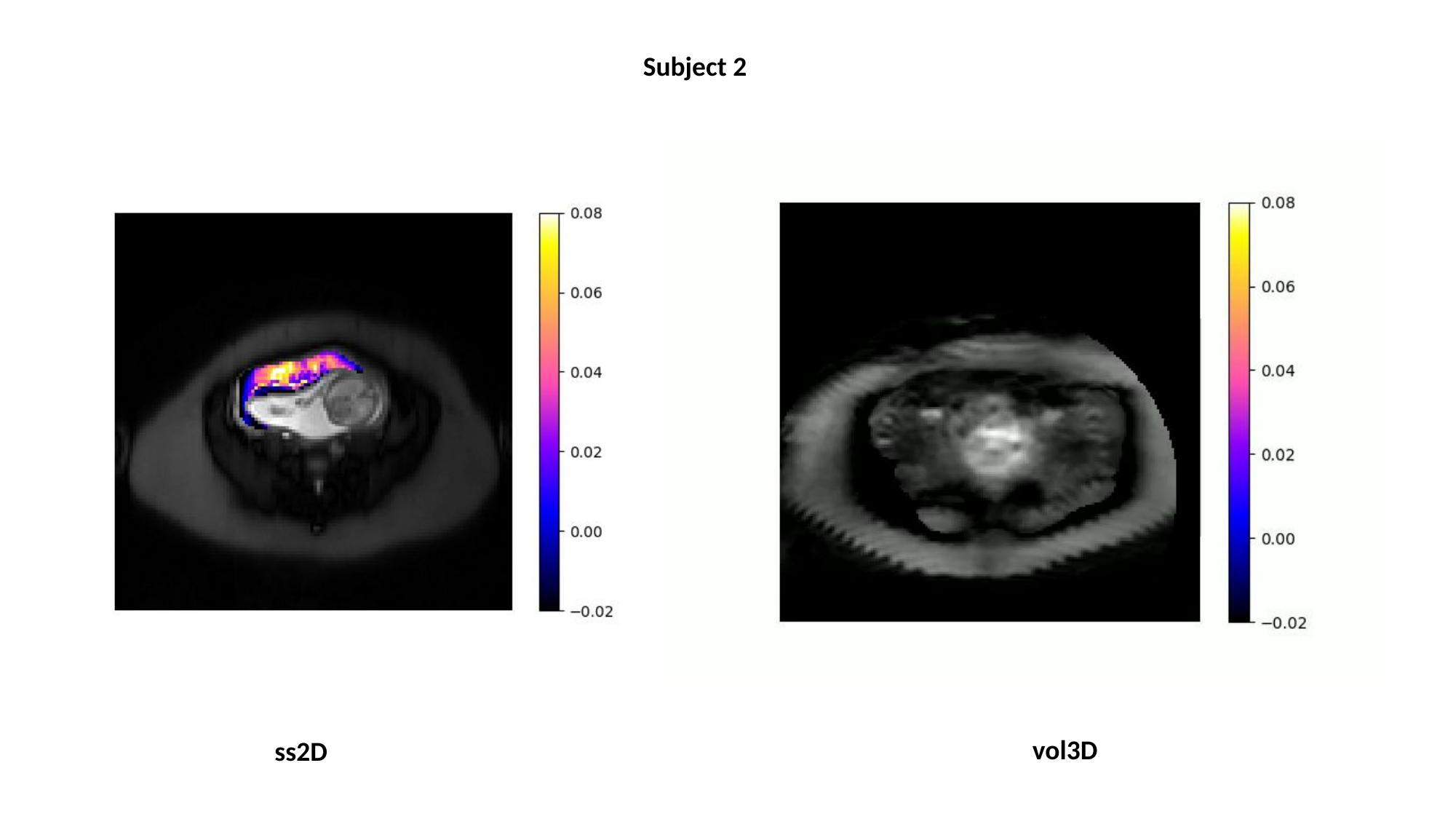

Subject 2
vol3D
ss2D

## Slide 4
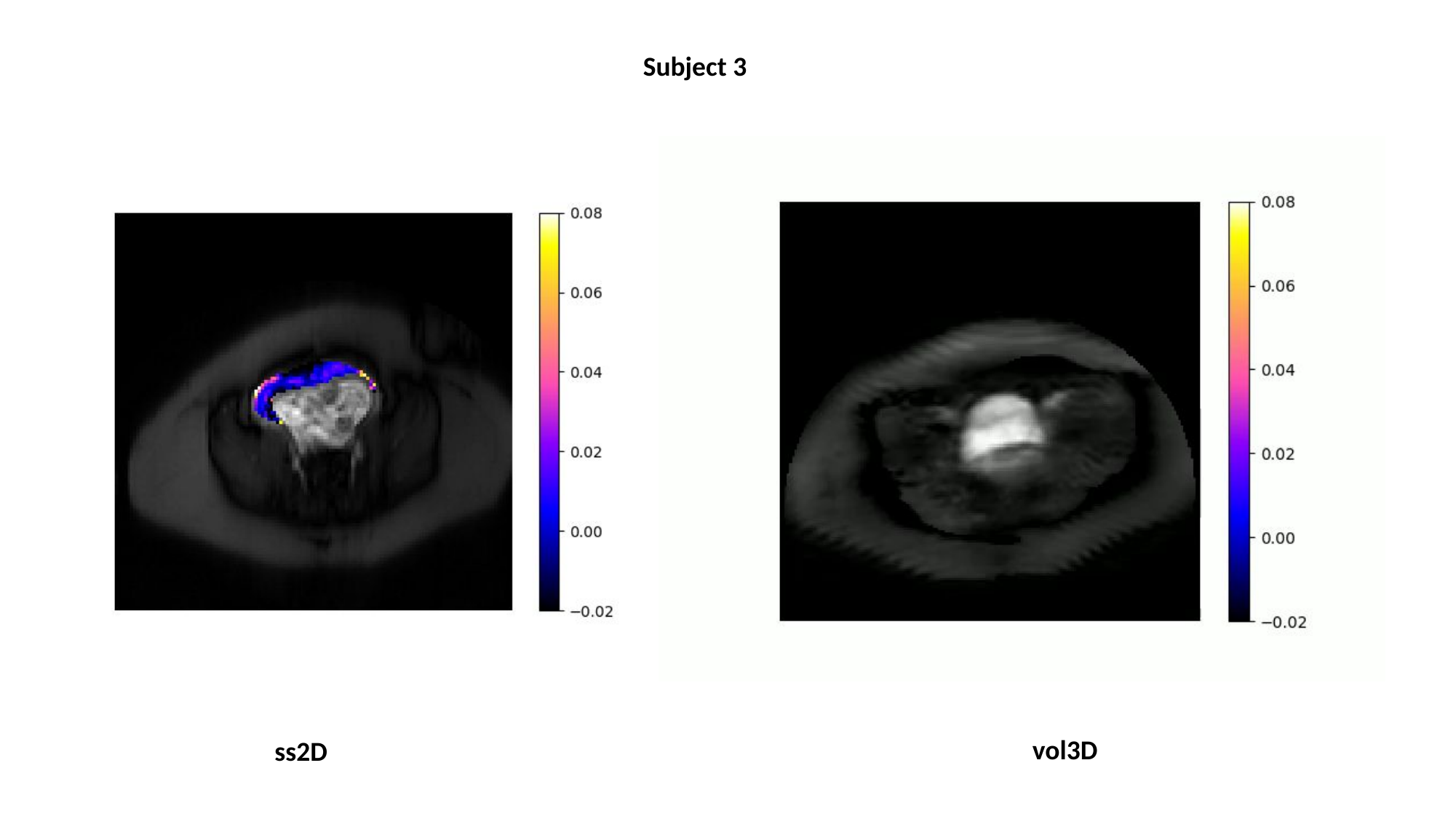

Subject 3
vol3D
ss2D

## Slide 5
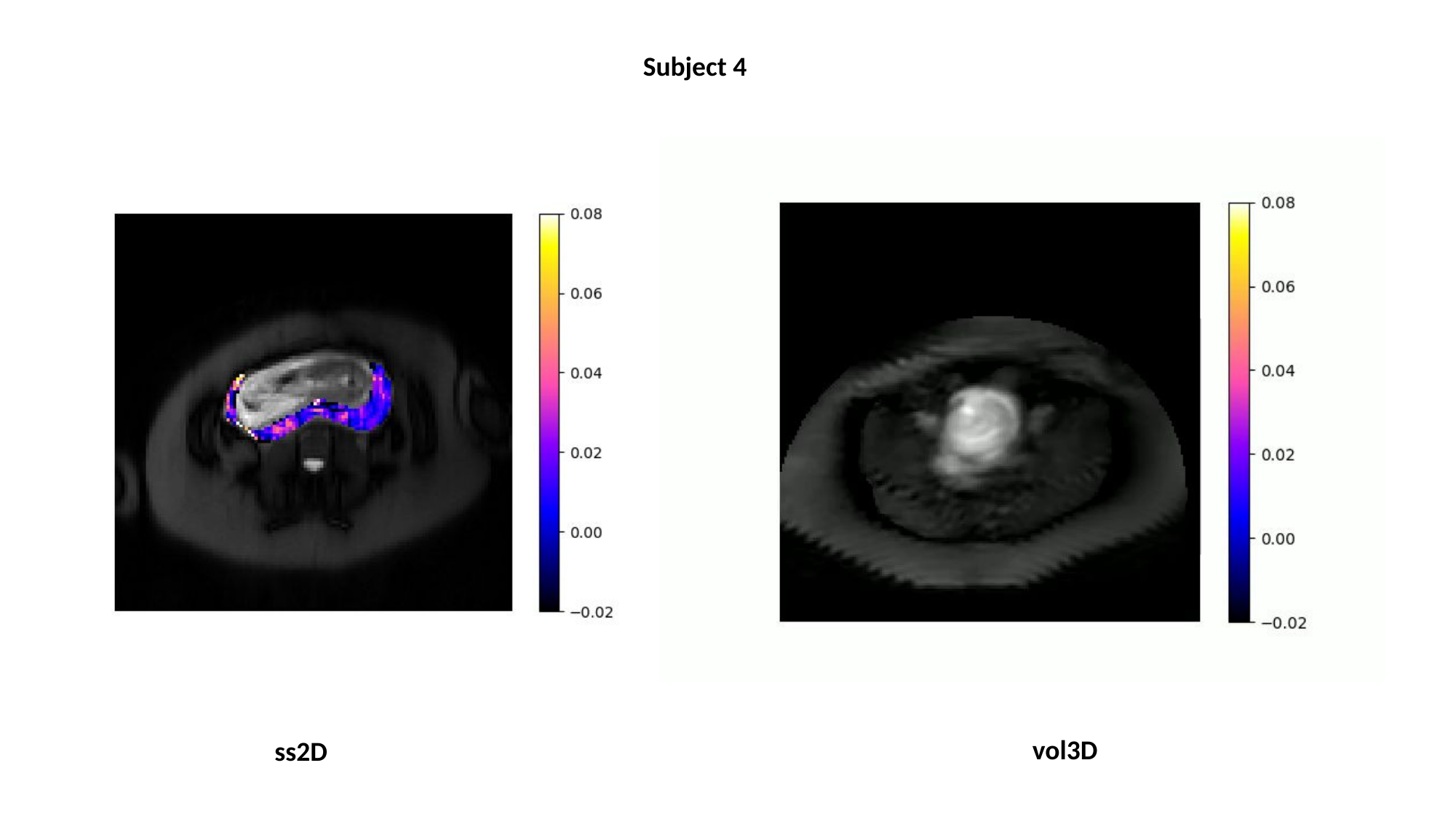

Subject 4
vol3D
ss2D

## Slide 6
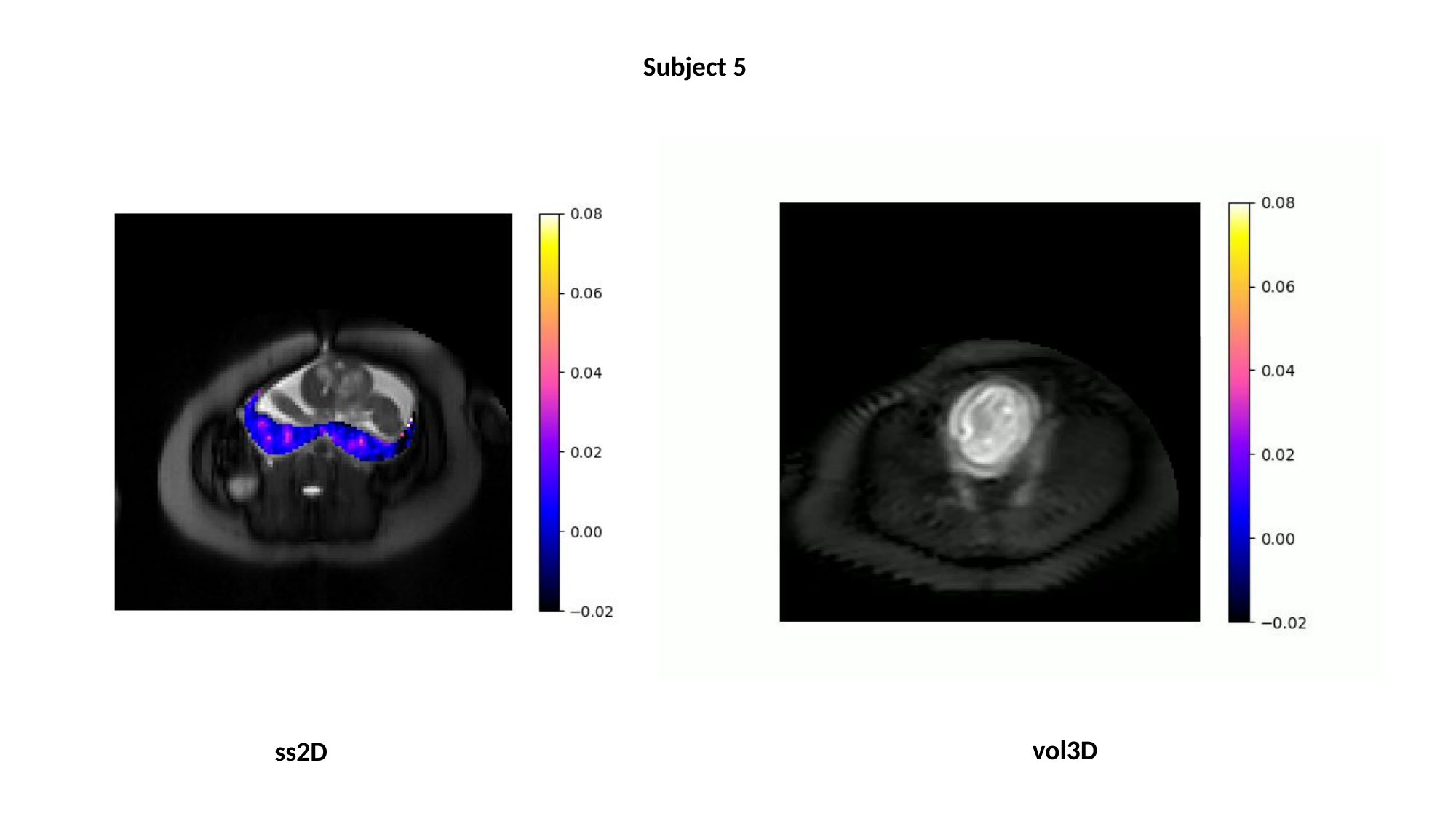

Subject 5
vol3D
ss2D

## Slide 7
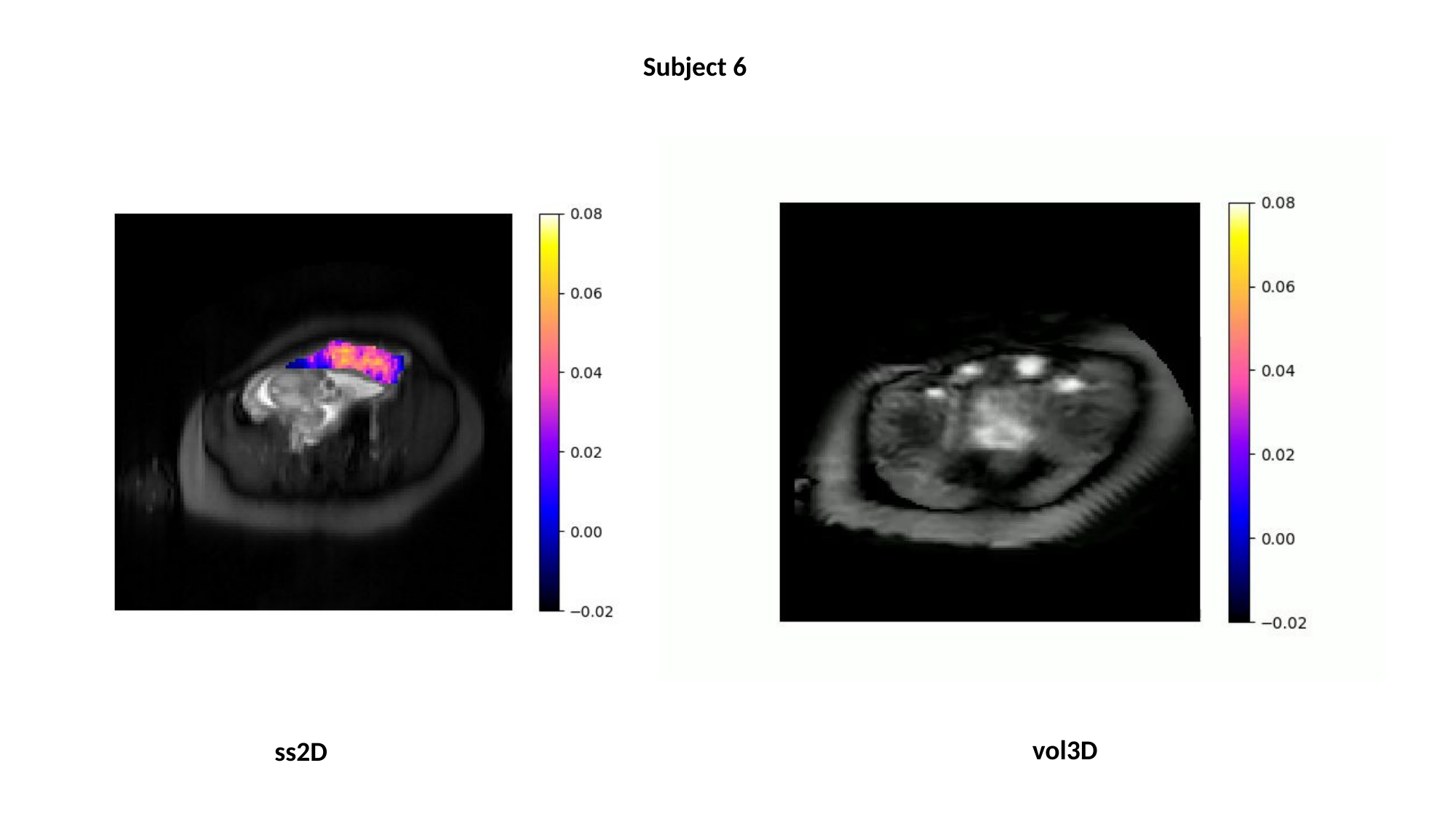

Subject 6
vol3D
ss2D

## Slide 8
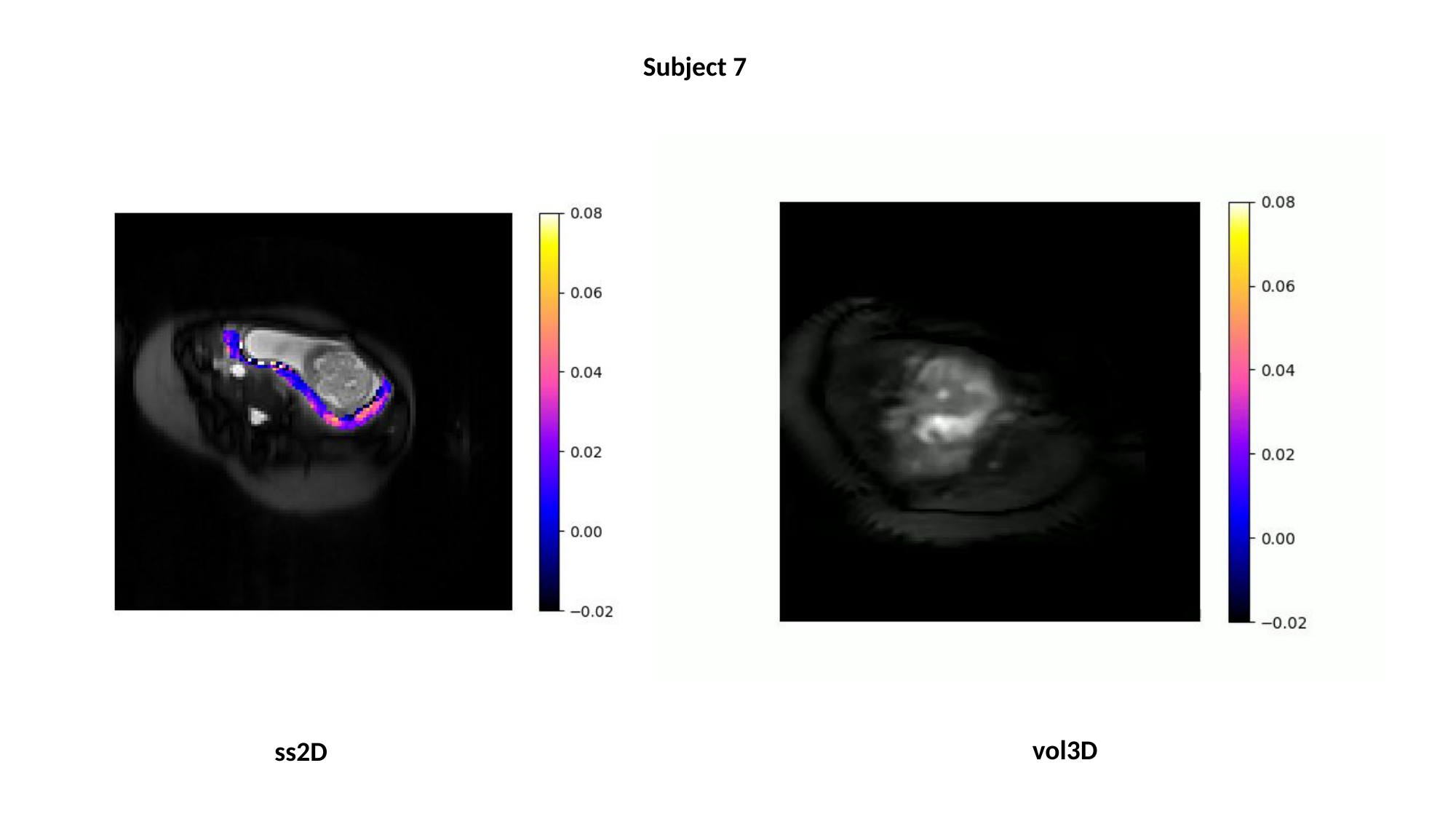

Subject 7
vol3D
ss2D

## Slide 9
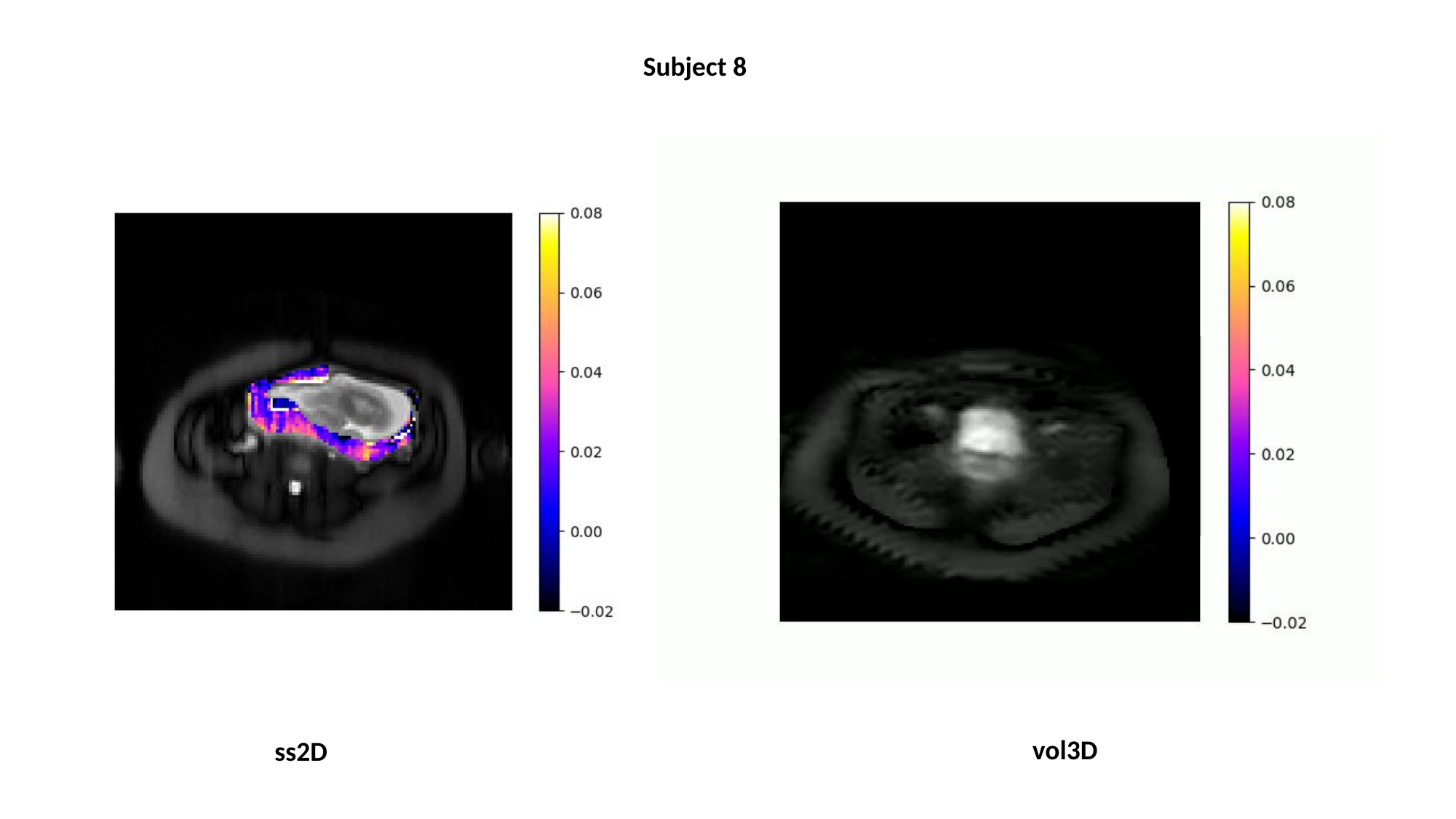

Subject 8
vol3D
ss2D

## Slide 10
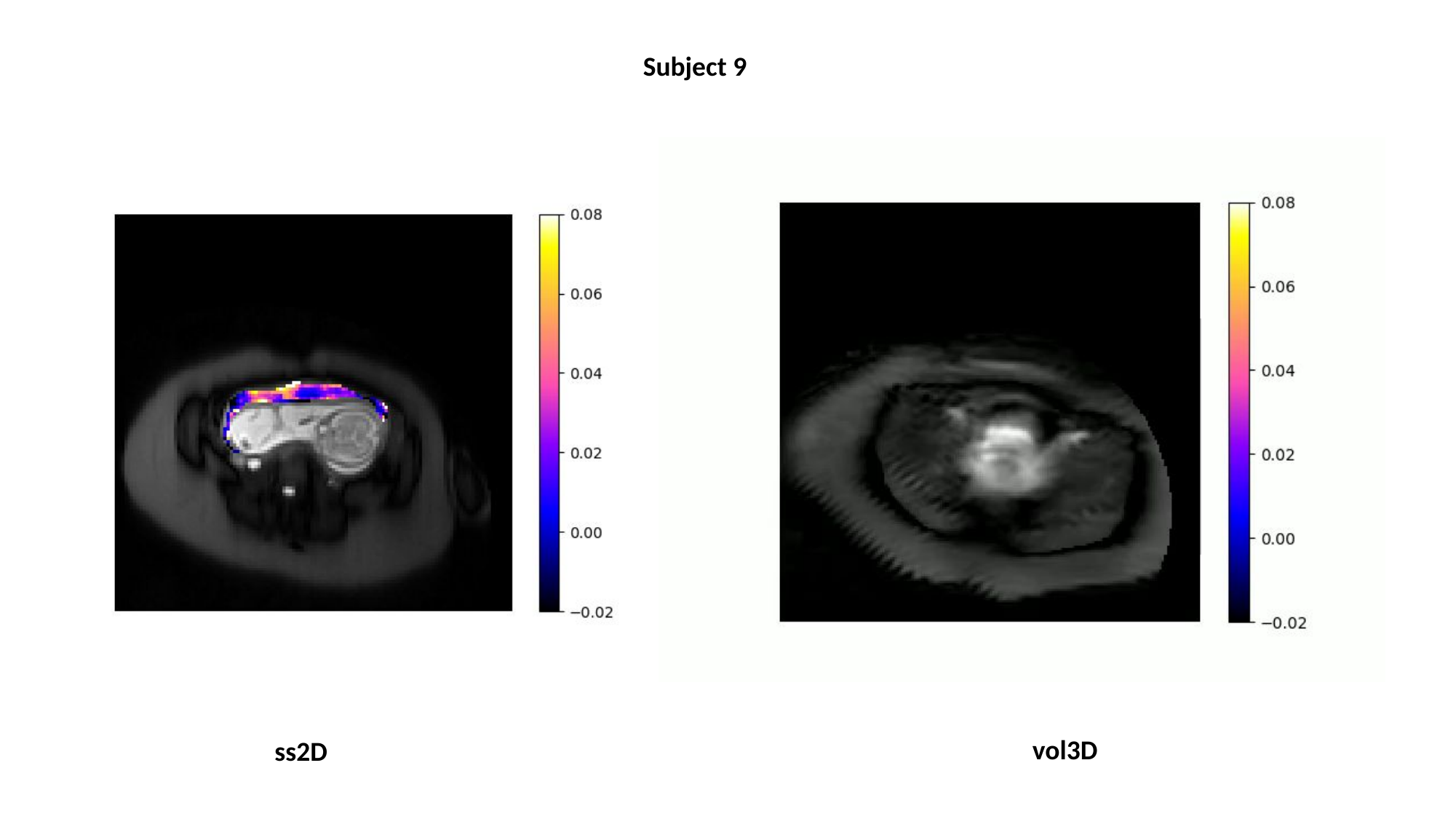

Subject 9
vol3D
ss2D

## Slide 11
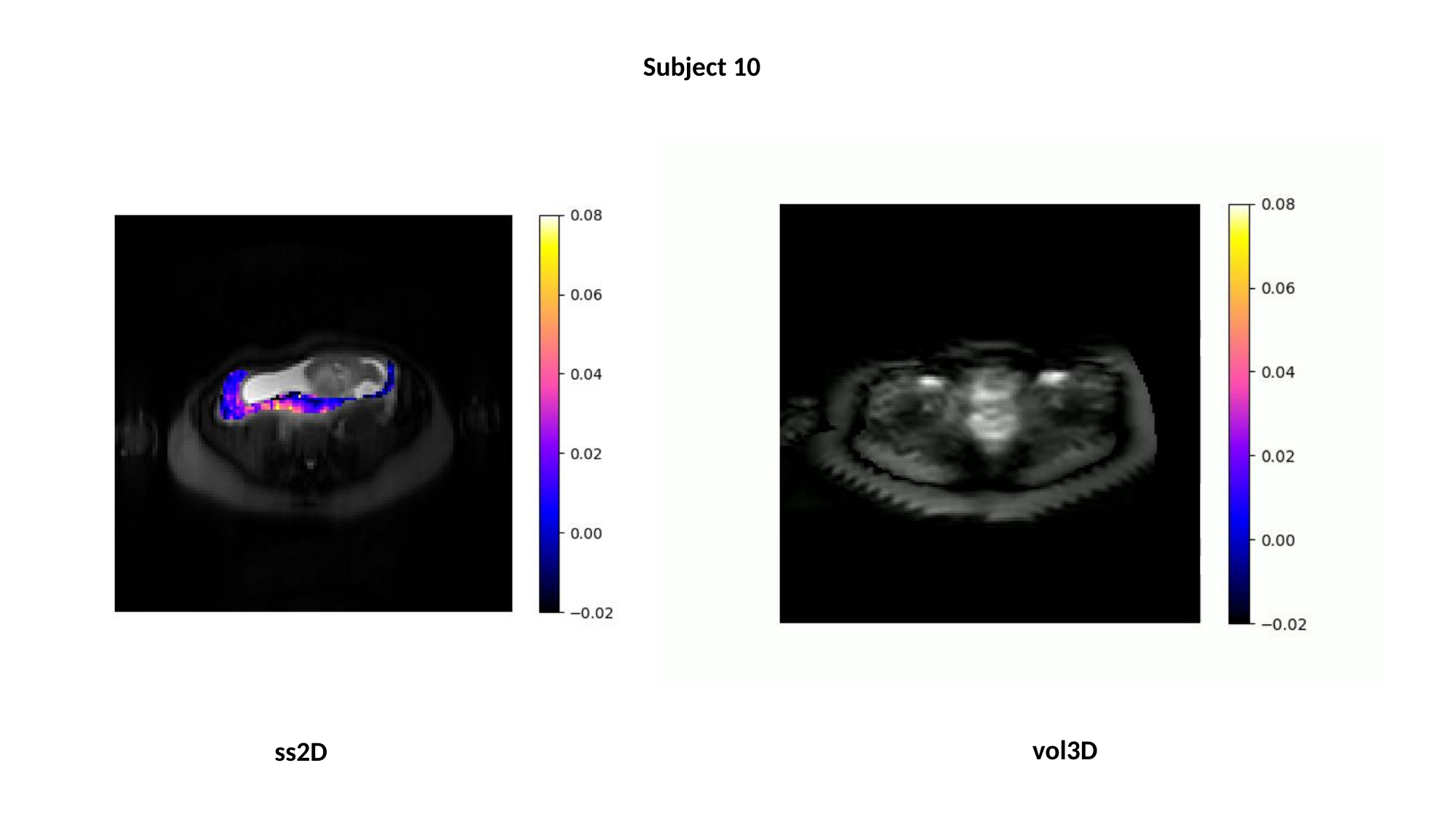

Subject 10
vol3D
ss2D

## Slide 12
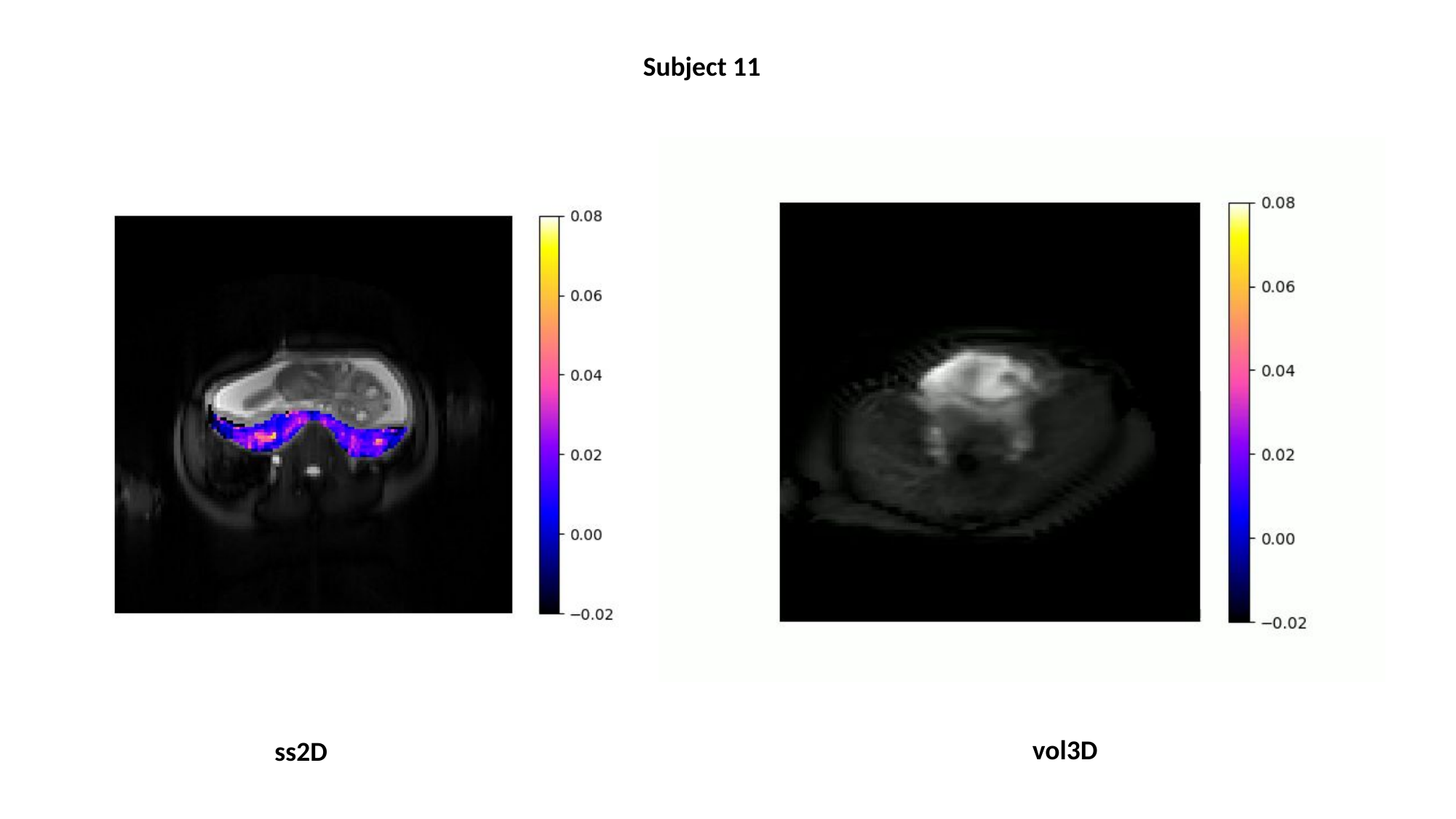

Subject 11
vol3D
ss2D

## Slide 13
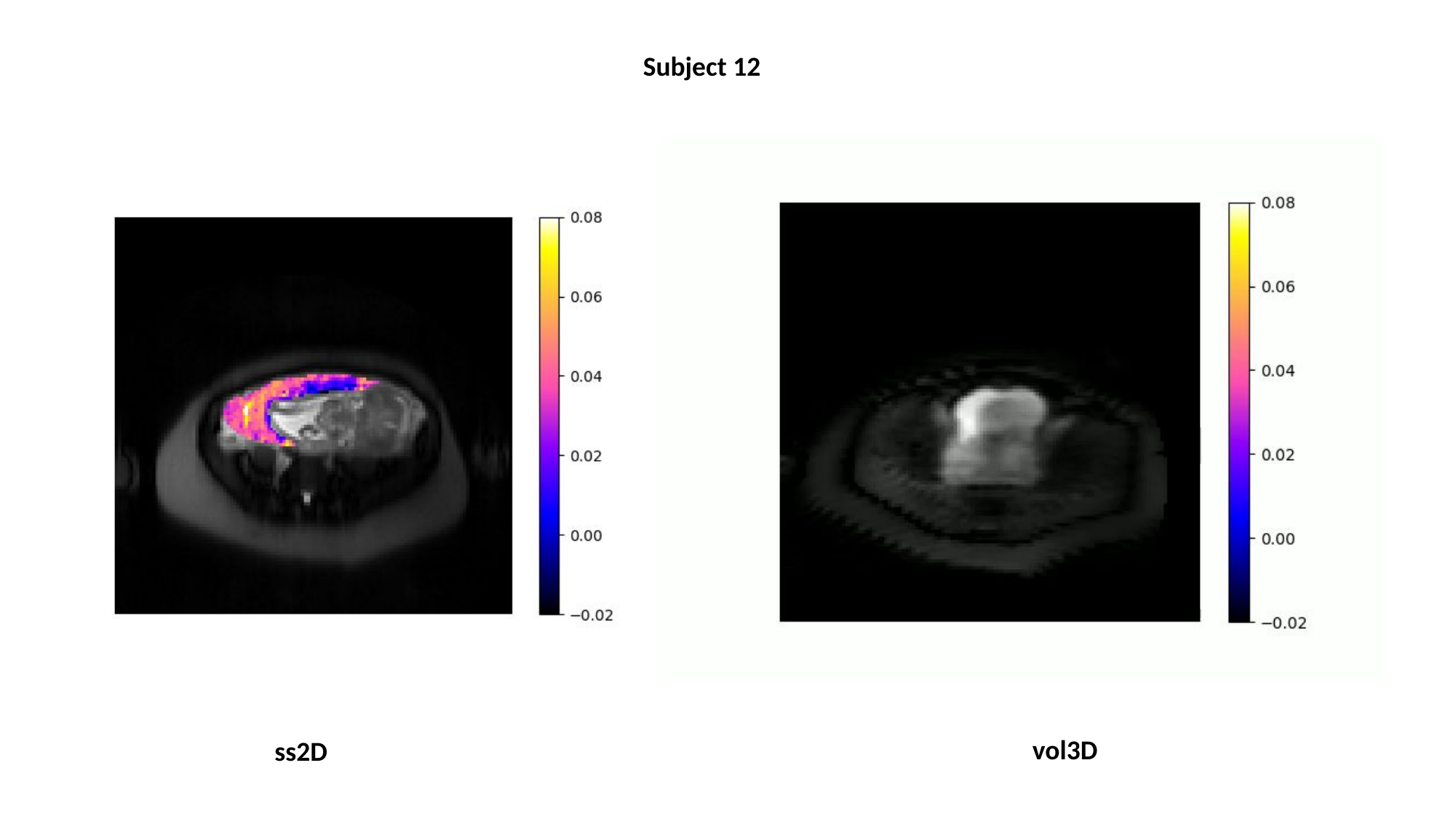

Subject 12
vol3D
ss2D
